# A New Method of Grafting Crowns upon the Roots of Teeth

**Published:** 1864-08

**Authors:** E. Collins

**Affiliations:** Richmond, Ind.


### A NEW METHOD OF GRAFTING CROWNS UPON THE ROOTS OF TEETH.

After fitting the tooth in the ordinary manner, drill out the fang two-thirds of its length with the largest size fang drill; then take a remer and enlarge the opening to near the outer margin of the fang. The enlargement should not extend up into the fang only about one-half the length of the canal made by the first drill. Clense out all foreign matter well and dry perfectly. Introduce a small pledget of lint, saturated with creosote, into the extremity of the canal. This done fill the root solidly with "Wood's Metal." Select a plain rubber tooth of the class, size and shade the case may require, fit it nicely upon the fang, and retain in position with wax. Cover the lateral face of the artificial crown, and collateral teeth with good plaster; when the plaster is sufficiently set, remove and let it dry for a few moments, then replace. Having removed all moisture from the previous filling, and parts adjacent, fill, with the above metal, in between the crown and root, the narrow spaces first; also, pack well around the roots. Then build on metal until the lingual face is made similar in form to the natural crown. Remove plaster and finish. This method of attaching crowns to roots, has the advantage over all others in the following particulars: The crown never requires resetting; the root is preserved from decay, and the tooth is as easily kept clean as the natural organs, hence no unpleasant effluvia arises therefrom, as is the case where teeth are inserted in the ordinary way. Try it and report progress.

E. COLLINS.

RICHMOND, IND.

